# Improving PMUT Receive Sensitivity via DC Bias and Piezoelectric Composition

**DOI:** 10.3390/s22155614

**Published:** 2022-07-27

**Authors:** Christopher Cheng, Travis Peters, Ajay Dangi, Sumit Agrawal, Haoyang Chen, Sri-Rajasekhar Kothapalli, Susan Trolier-McKinstry

**Affiliations:** 1 Department of Materials Science and Engineering, Penn State University, University Park, PA 16802, USA; cyc5@psu.edu (C.C.); tlp5454@psu.edu (T.P.); 2 Department of Biomedical Engineering, Penn State University, University Park, PA 16802, USA; dangi.ajay@gmail.com (A.D.); sua347@psu.edu (S.A.); haoyangchen@psu.edu (H.C.); szk416@psu.edu (S.-R.K.); 3 Department of Materials Science and Engineering and Materials Research Institute, Penn State University, University Park, PA 16802, USA

**Keywords:** PMUT, photoacoustics, receive sensitivity, DC bias

## Abstract

The receive sensitivity of lead zirconate titanate (PZT) piezoelectric micromachined ultrasound transducers (PMUTs) was improved by applying a DC bias during operation. The PMUT receive sensitivity is governed by the voltage piezoelectric coefficient, *h_31,f_*. With applied DC biases (up to 15 V) on a 2 μm PbZr_0.52_Ti_0.48_O_3_ film, e_31,f_ increased 1.6 times, permittivity decreased by a factor of 0.6, and the voltage coefficient increased by ~2.5 times. For released PMUT devices, the ultrasound receive sensitivity improved by 2.5 times and the photoacoustic signal improved 1.9 times with 15 V applied DC bias. B-mode photoacoustic imaging experiments showed that with DC bias, the PMUT received clearer photoacoustic signals from pencil leads at 4.3 cm, compared to 3.7 cm without DC bias.

## 1. Introduction

Miniaturized ultrasound sensors with low voltage operation and high transmit/receive signals are desirable for many applications. Micromachined ultrasound transducers prepared via MEMS fabrication have been developed to meet this need. Typically, their output acoustic signals are smaller than those from transducers built using bulk piezoelectrics [1]. A variety of methodologies have been utilized to improve the acoustic characteristics of MUTs, such as operating in the collapse mode for capacitive micromachined ultrasound transducers or fabricating dome-shaped membranes for piezoelectric micromachined ultrasound transducers [2,3].

Lead zirconate titanate and aluminum nitride are commonly used materials for piezoelectric micromachined ultrasound transducers. PZT is valued for its high piezoelectric coefficient and thus high transmit pressures at low actuation voltages [4]. The piezoelectric coefficient of aluminum nitride (AlN) is about an order of magnitude lower than PZT, and its dielectric constant is approximately two orders of magnitude lower than that of PZT [5,6]. Although the receive sensitivity of AlN is desirable, higher transmit power is needed for most ultrasonic imaging.

Receive sensitivity is a function of the piezoelectric voltage coefficient, *h_31,f_*, which is expressed as:

*h_31,f_* = *e_31,f_*/*ε_r_* (1)

where *e_31,f_* and *ɛ_r_* are the piezoelectric coefficient and permittivity, respectively. Thus, to improve receive sensitivity, the piezoelectric coefficient must be high and/or the permittivity must be low.

When large electrical fields are applied to a ferroelectric, such as PZT, the permittivity decreases below the zero field value due to reduced intrinsic and extrinsic contributions to the permittivity [7]. Moreover, the piezoelectric coefficient may also increase with large DC electric fields, as domains more readily remain aligned with the applied electric field [8,9,10,11]. Thus, if a DC electric field is applied while the PMUT functions as a receiver, the receive sensitivity should be higher than at zero applied bias. The trends in piezoelectric coefficient and permittivity have been reported for air-coupled transducers [6] but have not been demonstrated in underwater ultrasonic and photoacoustic applications.

In addition, this paper discusses different PZT compositions and their suitability for ultrasound receive sensitivity. The morphotropic phase boundary (MPB) composition of PZT with a Zr/Ti ratio of 52/48 is often utilized for its high permittivity and piezoelectric coefficients [12]. As the composition moves off the MPB, the piezoelectric coefficient and permittivity decrease [12,13] at different rates. Thus, on changing the composition, the permittivity may decrease faster than that of the piezoelectric coefficient. It is hypothesized that for ultrasound applications moving off the MPB may result in higher receive sensitivity while maintaining relatively high transmit power.

## 2. Materials and Methods

To test whether the permittivity and piezoelectric coefficient increase with DC bias, unreleased samples with an architecture similar to that of PMUTs were fabricated. A 500 μm Si wafer with 1 μm of SiO_2_ on both sides (NOVA Electronic Materials, TX, USA) was used as the substrate. The bottom electrode was deposited by sputtering 30 nm of Ti, annealing it at 700 °C for 15 min with 10 sccm of oxygen flow, and then sputtering 100 nm of Pt at a substrate temperature of 500 °C [14]. To facilitate orientation of the piezoelectric film, a PZT seed layer (Mitsubishi Materials Corporation, Hyogo, Japan) was spun, pyrolyzed, and crystallized, as described elsewhere [10,15,16,17]. For the MPB composition, 14 mol% lead excess Pb(Zr_0.52_Ti_0.48_)O_3_ solution doped with 2% Nb (Mitsubishi Materials Corporation, Hyogo, Japan) was spun on at 2750 RPM for 45 s. The film was pyrolyzed at 100 °C for 1 min and 300 °C for 4 min, followed by crystallization in a lead-rich rapid thermal annealer for 1 min at 700 °C. This process was repeated ~20 times until a film thickness of ~2.0 μm was achieved.

In order to investigate various compositions, an inverted mixing order process was used to prepare PZT solutions with different Zr/Ti ratios [18,19,20,21,22,23,24]. 0.4 M solutions of PZT with Zr/Ti ratios of 52/48, 40/60, 30/70, and 20/80 were spun on the hot-sputtered Pt substrates with the Mitsubishi seed layer at 3000 RPM for 30 s. The film was pyrolyzed at 250 °C for 30 s and 400 °C for 1 min, followed by crystallization in a lead-rich rapid thermal annealer for 2 min at 650 °C. The top Pt electrodes were patterned via liftoff and annealed at 600 °C for 1 min. These samples were then diced. Pieces were adhered to the center of a Si wafer, and strain gauges were superglued onto the die. The *e_31,f_* was measured via the wafer flexure method [25].

In addition, 14 mol% lead excess Pb(Zr_0.52_Ti_0.48_)O_3_ solution doped with 2% Nb (Mitsubishi Materials Corporation, Hyogo, Japan) was spun on a separate wafer at 2750 rpm for 45 s. The film was pyrolyzed at 100 °C for 1 min and 300 °C for 4 min, followed by crystallization in a lead-rich rapid thermal annealer for 1 min at 700 °C. This process was repeated ~20 times until a total thickness of ~2.0 µm was achieved. The resulting film had a {100} Lotgering factor >97%. The top Pt electrodes were patterned via liftoff and annealed at 600 °C for 1 min. The wafer was then diced and clamped to the center of a Si carrier wafer, and strain gauges were glued onto the die.

Following sample fabrication, the effect of DC bias on *e_31,f_* and *ε_r_* was measured on clamped samples. Prior to measurement, each device was poled at 35 V for 20 min at 150 °C. Pre-poling allowed the DC bias dependence to be deconvoluted from poling, as was apparent in previous studies. The wafer flexure method was used to characterize *e_31,f_*.

The design, fabrication process, and characterization for circular 6–8 MHz PMUTs are described elsewhere [10,17]. The PMUTs were wire-bonded to a pin grid array (PGA) or a circuit board and coated with 2 µm of parylene for waterproofing.

**Figure 1:**
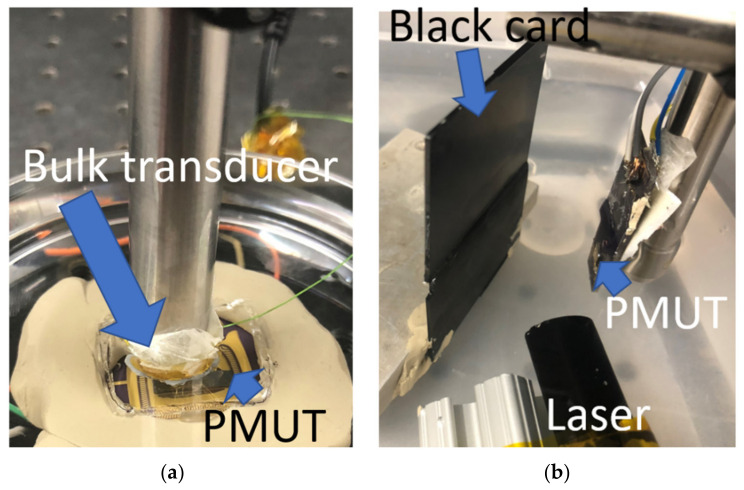
Experimental setup for (**a**) acoustic and (**b**) photoacoustic measurements. For pitch catch measurements, the bulk transducer source in (**a**) was replaced with a glass slide.

The receive sensitivity was obtained with the setup in Figure 1a. The bulk transducer, functioning as a pressure source, was excited using a unipolar 5 V_pp_ burst sine waveform at its resonance, 3.5 MHz, 8.5 mm distance from the receiving PMUT.

For photoacoustic measurements, a PMUT was submerged in a distilled water tank facing a black card, as seen in Figure 1b. A tunable (680–980 nm) nanosecond laser (Phocus Mobile, Opotek Inc., Carlsbad, CA, USA) delivered 10 ns pulse width laser pulses at a repetition frequency of 10 Hz and 120 mJ/pulse energy through a custom-fabricated fiber optic bundle (Fiberoptic Systems Inc., Simi Valley, CA, USA). The PMUT was housed in a custom printed circuit board and optical fiber bundle apparatus with an additional external gain of 20 dB as described elsewhere [10,17]. Pencil leads 0.3 mm in diameter were suspended at different heights in an agar-gel phantom to test the photoacoustic sensitivity of the PMUT as a function of depth and to see if DC biasing produced improved depth sensitivity. The preparation of the agar-gel phantom is described elsewhere [17]. A single element of the PMUT was linearly scanned across the fixed target, and the photoacoustic A-lines were compiled to form a B-mode photoacoustic image.

For pitch-catch measurements, one PMUT element functioned as the ultrasound transmitter, and an adjacent element on the same PMUT device functioned as a receiver. The ultrasound waves travel from the transmitter and, at a defined distance, reflect at an area of high acoustic impedance mismatch. The waves then travel back to the device, where the signal is acquired by the receiver. In this setup, the PMUT was placed inside a pin-grid array cavity, which was filled with distilled water. A glass slide was placed on top of the cavity, acting as a reflector. One PMUT element was excited with a single cycle of unipolar 5 V_pp_ in a sinusoidal burst mode. Another PMUT element functioned as a receiver with an external 39 dB gain. A bias tee was used to apply a DC bias to the PMUT during receive.

## 3. Results

### 3.1. Structural Measurements

**Figure 2:**
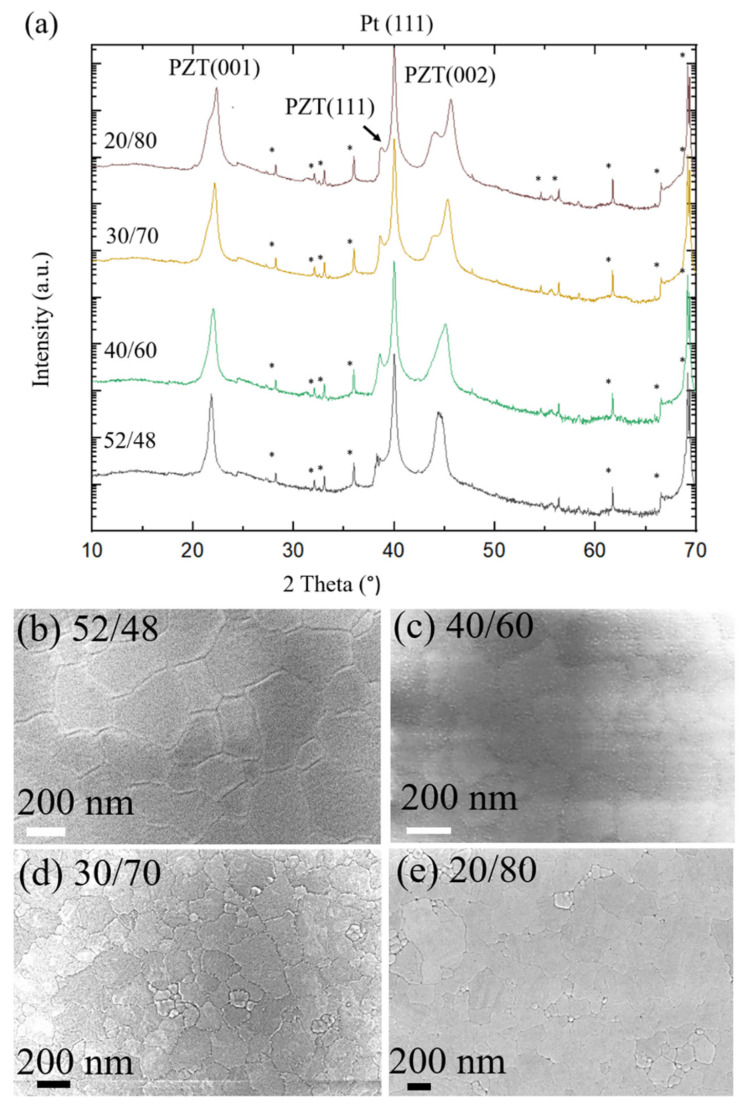
Structural characterization of fabricated samples of various PZT compositions (given as Zr/Ti ratio). (**a**) XRD analysis and (**b**–**e**) are surface FESEM images. (* = substrate peaks).

The X-Ray diffraction patterns and FESEM images of various PZT compositions are shown in Figure 2. The Lotgering factors are similar and show highly oriented, phase pure perovskite PZT for each composition. As expected, the degree of tetragonal splitting increases as the film becomes more Ti rich. In addition, the grain sizes were determined using the line intercept method. The grain sizes for 52/48, 40/60, 30/70, and 20/80 were 212 ± 19 nm, 203 ± 113 nm, 197 ± 24 nm, and 232 ± 26 nm, respectively, indicating statistically similar grain sizes for all compositions.

### 3.2. Electrical Measurements

**Figure 3:**
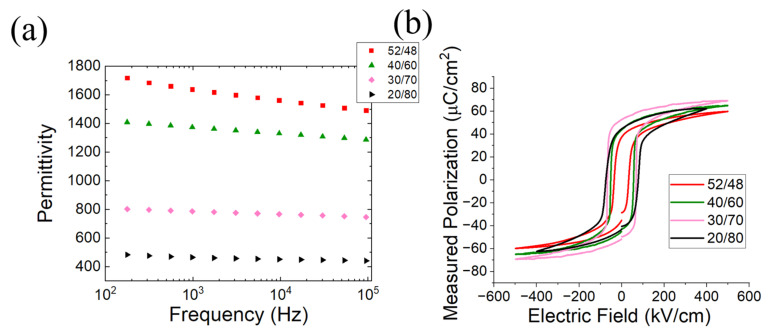
(**a**) Permittivity vs. frequency and (**b**) hysteresis loops of various PZT compositions.

The baseline dielectric characterization of the films is given in Figure 3. As expected, the permittivity decreased for compositions of the morphotropic phase boundary (MPB). As the composition approaches lead titanate, the remanent polarization increases due to increased tetragonality and possibly, a reduction in the film stress. It should be noted that for the 20/80 composition, a maximum of 400 kV/cm was applied for the hysteresis loop measurement, as the film becomes leaky at greater electric fields, as documented in the literature [12].

**Figure 4:**
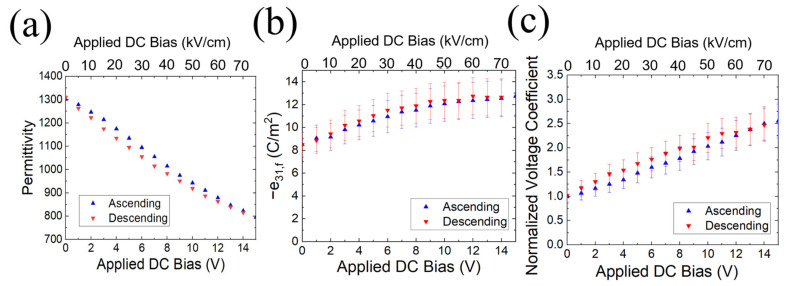
(**a**) Permittivity (**b**) *e_31,f_* piezoelectric coefficient, and (**c**) normalized *h_31,f_* coefficient as a function of DC bias with various PZT compositions.

Figure 4 shows the permittivity (*ɛ_r_*) and piezoelectric coefficient (*e_31,f_*) as a function of DC bias for the hot-poled PZT 52/48 film. At 15 V (75 kV/cm), the permittivity decreases by a factor of 0.6, and the piezoelectric coefficient increases by a factor of 1.7 relative to the zero bias values. The *e_31,f_* starts to saturate at ~10 V, corresponding to the value of the coercive field ~50 kV/cm. Based on the normalized voltage coefficient, the receive sensitivity is expected to improve by ~2.5 times with a DC bias of 15 V.

**Figure 5:**
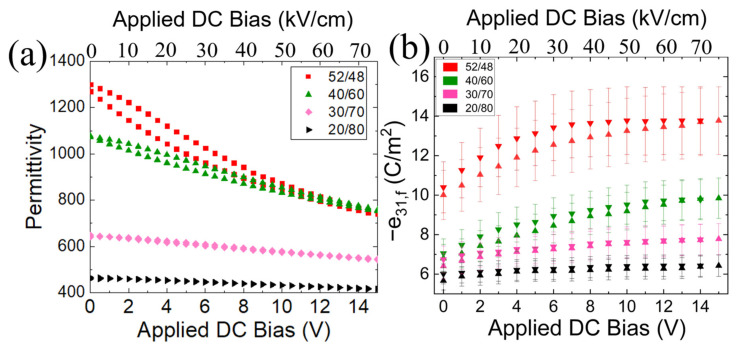
(**a**) Permittivity and (**b**) piezoelectric coefficient, as a function of DC bias for various average PZT film compositions.

Figure 5 shows the tunability of the different PZT compositions. The dielectric tunabilities for the 52/48, 40/60, 30/70, and 20/80 compositions were 43%, 30%, 16%, and 11%, respectively, at an electric field of 75 kV/cm. The PZT 52/48 had the largest piezoelectric and permittivity tunabilities, as expected, since the maximum polarizability occurs at the brink of structural instability (i.e., at the MPB). The decrease in the permittivity and piezoelectric coefficient as a function of composition was different. From the MPB to the PZT 20/80 film, the zero bias permittivity and piezoelectric coefficient decreased by a factor of 65% and 43%, respectively.

**Figure 6:**
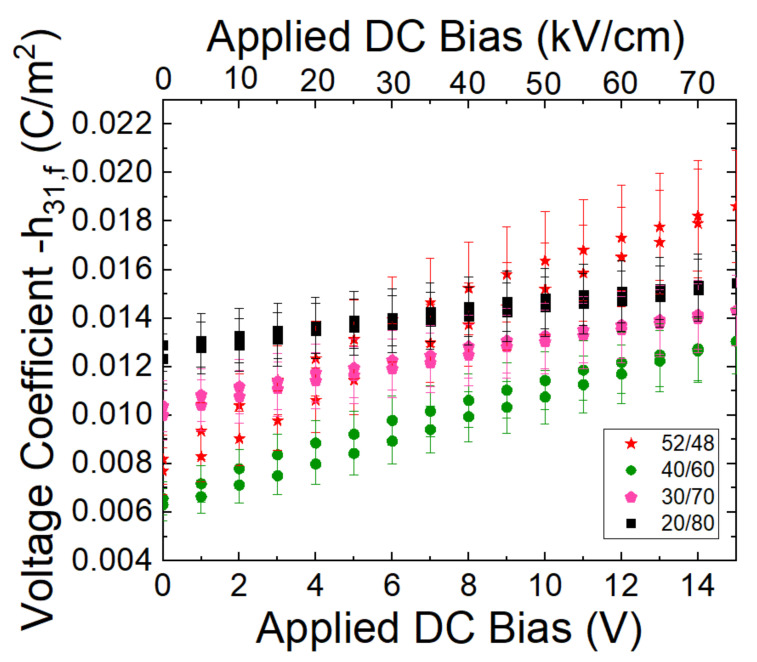
Voltage coefficient versus DC bias for various PZT compositions.

The resulting voltage coefficient, which controls the receive sensitivity, is given in Figure 6. For operation at 0 DC bias, PZT 20/80 has a higher voltage coefficient than the other compositions. However, when a DC bias of 75 kV/cm is applied, the voltage coefficient of a 52/48 composition can match and potentially surpass the voltage coefficient of the PZT 20/80 composition.

### 3.3. Underwater Acoustic Measurements

**Figure 7:**
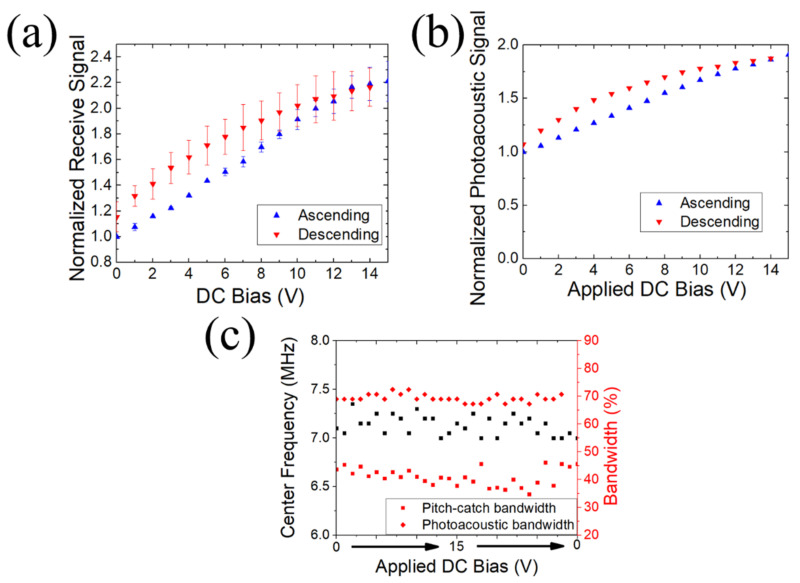
(**a**) Ultrasound receive signal and (**b**) normalized photoacoustic signal as a function of DC bias based on setups in Figure 1a,b, respectively. (**c**) indicates the center frequency (black) and bandwidths (red) as a function of DC bias. The device used for these was PZT 52/48 with 2 μm thickness.

The underwater receive sensitivity and photoacoustic sensitivity as a function of DC bias are shown in Figure 7 for a PMUT made with MPB PZT. The ultrasound receive sensitivity and the photoacoustic receive signal increased by approximately two times, agreeing with the electrical measurements in Figure 6. With no DC bias, the *e_31,f_* and the permittivity values were –10 C/m^2^ and 1300, respectively, with *h_31,f_* = −0.0062 C/m^2^. At 15 V DC bias, the *e_31,f_* and the relative permittivity were −13.8 C/m^2^ and 740, respectively, yielding *h_31,f_* = −0.018 C/m^2^. This is ~18% of the voltage coefficient of AlN, which has a piezoelectric coefficient and permittivity of −1.05 C/m^2^ and 10.5, respectively [5,6].

It has been reported that the resonant frequencies and bandwidth can increase as much as 33% with an applied DC bias for air-coupled transducers [6]. This may be a consequence of the applied voltage increasing the in-plane stress of the piezoelectric. This would occur in the case when stress dominates over the flexural rigidity, such that increased stress increases the resonant frequency [26]. To examine whether significant changes can be seen in operating frequency and bandwidth as a function of DC bias for these underwater PMUT, the center frequency and bandwidth were extracted from the Fourier transform of the pitch-catch and photoacoustic receive signals, as shown in Figure 7c. In both cases, the center frequency (6.89 MHz) and bandwidth remained relatively constant as a function of DC bias (from 0–15 V). The center frequency had a standard deviation of 0.6%. The photoacoustic bandwidth and pitch catch bandwidths had standard deviations of 2% and 8%, respectively. The photoacoustic center frequencies and bandwidth also showed little variation with DC bias, albeit the photoacoustic bandwidth was higher (69%) than the pitch-catch bandwidth (41%). The higher bandwidth for photoacoustics compared to pitch-catch is due to two factors: First, in pitch-catch ultrasound imaging, spatial resolution is governed by the frequency of the transmitted acoustic wave arriving at the receiver, whereas in photoacoustic imaging, the laser pulses have nanosecond-scale excitation with much wider frequency content [27,28]. Second, the bandwidth for one-direction sound propagation is larger than the two-way bandwidth.

**Figure 8:**
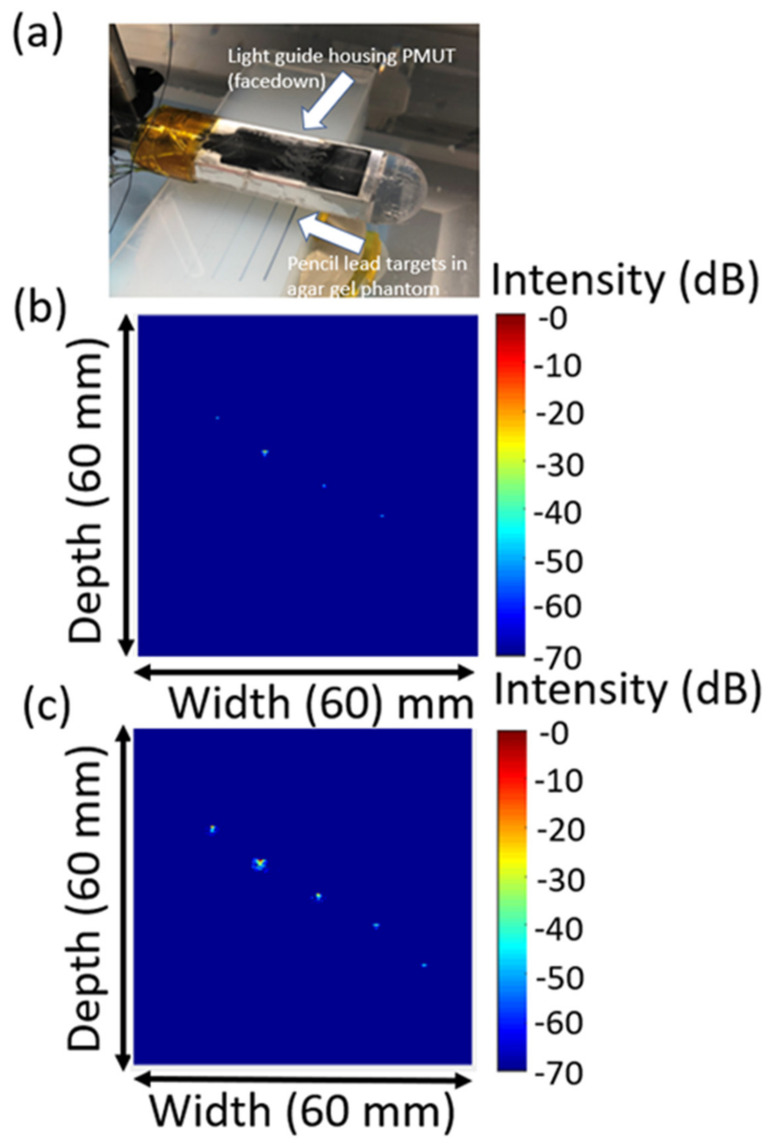
(**a**) Picture of the light-guide fiber optic assembly housing the PMUT. The PMUT is facing downward at the agar gel phantom with pencil leads of varying heights. The resulting images are with (**b**) DC bias and (**c**) 15 V DC bias operation (75 kV/cm) for 52/48 composition. Log compression was done together with (**b**,**c**) for comparison.

**Figure 9:**
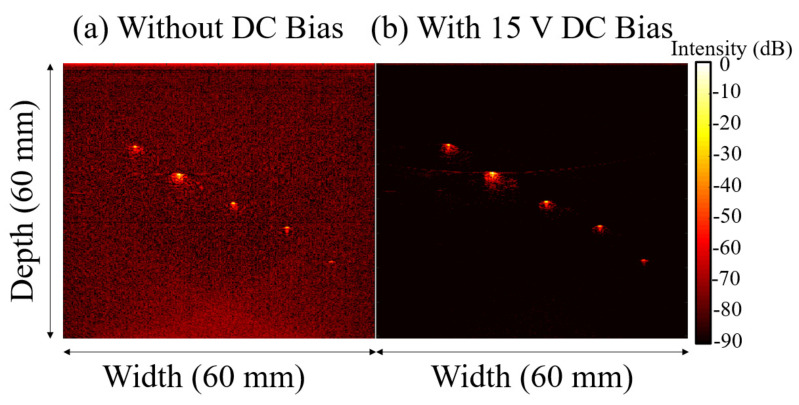
Images of Figure 8 where log compression was done separately for images (**a**) without DC bias and (**b**) with DC bias.

B-mode photoacoustic images were reconstructed from the raw voltage-time data, as shown in Figure 8 and Figure 9. Without DC bias, a clear photoacoustic signal was only obtained from four of the five pencil leads, the fourth being at ~3.7 cm in depth. With 15 V DC bias, the photoacoustic signal was increased for all pencil lead targets. In addition, the PMUT was able to receive a photoacoustic signal from the fifth pencil lead at 4.3 cm depth. Figure 8 shows an ~20 dB improvement in the image signal. The cause of this is that images with and without DC bias were subjected to log compression together in the same data matrix, e.g., the raw data for Figure 8b,c were subjected to one log compression operation for overall image comparison. Thus, when compressing the two images in a single data matrix, the normalization caused the observed signal intensity difference between the two images to exceed two times. When the log compression was done separately for each image, as shown in Figure 9, the photoacoustic signal with DC bias was ~2 times greater than the signals seen without DC bias, agreeing with the electrical measurements. It is observed that the signal-to-noise ratio (SNR) was significantly better with DC bias than without DC bias and applying the log compression of both images in the same dataset caused a larger improvement in the imaging signal and quality. It is not clear why this should be the case.

## 4. Conclusions

The receive sensitivities increased by ~2 times when 15 V (75 kV/cm) was applied to the MPB PZT PMUT, due to the increase in the *e_31,f_* and a decrease in the relative permittivity. Operating at a 15 V DC bias also yielded stronger photoacoustic signals and allowed detection of signals at greater depths compared to without DC bias. When no DC bias is applied, PZT with composition 20/80 has a significantly higher voltage coefficient than other PZT compositions tested. When 15 V DC bias (75 kV/cm) is applied, PZT 52/48 matched the receive sensitivity of PZT 20/80. Thus, operating at higher electric fields in piezoelectric-based thin film devices can improve sensitivities. It is likely that a comparable improvement could be achieved by imprinting the PZT. This would be useful as it would allow higher receive sensitivities for the PMUT without the need for a DC bias on receive.

It is known that higher applied DC electric fields result in a shorter lifetime and faster appearance of electrothermal breakdown events [29,30,31]. In practice, the times for which the DC voltage would need to be applied are quite short (microsecond time scales per pulse), so it is unlikely that the PMUT lifetime will be limited by the DC bias based on previously reported highly accelerated lifetime data. However, it is also known that the lifetime and field-induced deflection can further be reduced by combinations of humidity and AC voltage [32]. Thus, the time-dependence of the receive sensitivity at different DC biases in the waterproofed PMUT should be explored while the device is submerged in future work.

## Data Availability

The corresponding author will provide data available on reasonable request due to restrictions. The data are not publicly available due to funding agent policies.

## References

[1] Mattiat O.E. (1971). Ultrasonic Transducer Materials.

[2] Park K.K., Oralkan O., Khuri-Yakub B.T. (2013). A Comparison Between Conventional and Collapse-Mode Capacitive Micromachined Ultrasonic Transducers in 10-MHz 1-D Arrays. IEEE Trans. Ultrason. Ferroelectr. Freq. Control..

[3] Hajati A., Latev D., Gardner D., Hajati A., Imai D., Torrey M., Schoeppler M. (2012). Three-Dimensional Micro Electromechanical System Piezoelectric Ultrasound Transducer. Appl. Phys. Lett..

[4] Kim K., Choi H. (2021). High-Efficiency High-Voltage Class F Amplifier for High-Frequency Wireless Ultrasound Systems. PLoS ONE.

[5] Wang Q., Lu Y., Mishin S., Oshmyansky Y., Horsley D.A. (2017). Design, Fabrication, and Characterization of Scandium Aluminum Nitride-Based Piezoelectric Micromachined Ultrasonic Transducers. J. Microelectromech. Syst..

[6] Kusano Y., Wang Q., Luo G.L., Lu Y., Rudy R.Q., Polcawich R.G., Horsley D.A. (2018). Effects of DC Bias Tuning on Air-Coupled PZT Piezoelectric Micromachined Ultrasonic Transducers. J. Microelectromech. Syst..

[7] Bassiri-Gharb N., Fujii I., Hong E., Trolier-McKinstry S., Taylor D., Damjanovic D. (2007). Domain Wall Contributions to the Properties of Piezoelectric Thin Films. J. Electroceram..

[8] Muralt P., Ledermann N., Baborowski J., Barzegar A., Gentil S., Belgacem B., Petitgrand S., Bosseboeuf A., Setter N. (2005). Piezoelectric Micromachined Ultrasonic Transducers Based on PZT Thin Films. IEEE Trans. Ultrason. Ferroelectr. Freq. Control..

[9] Masuda Y., Baba A.D.C. (1985). Bias and Frequency Dependence of the Dielectric Constant PZT Family Ferroelectric Ceramics. Jpn. J. Appl. Phys..

[10] Cheng C.Y., Dangi A., Ren L., Tiwari S., Benoit R.R., Qiu Y., Lay H.S., Agrawal S., Pratap R., Kothapalli S.R. (2019). Thin Film PZT-Based PMUT Arrays for Deterministic Particle Manipulation. IEEE Trans. Ultrason. Ferroelectr. Freq. Control..

[11] Dausch D.E., Gilchrist K.H., Carlson J.R., Castelucci J.B., Chou D.R., von Ramm O.T. Improved Pulse-Echo Imaging Performance for Flexure-Mode pMUT Arrays. Proceedings of the 2010 IEEE International Ultrasonics Symposium.

[12] Hiboux S., Muralt P., Maeder T. (1999). Domain and Lattice Contributions to Dielectric and Piezoelectric Properties of Pb(Zr_x_,Ti_1-x_)O_3_ Thin Films as a Function of Composition. J. Mater. Res..

[13] Gerber P., Bottger U., Waser R. (2006). Composition Influences on the Electrical and Electromechanical Properties of Lead Zirconate Titanate Thin Films. J. Appl. Phys..

[14] Fox A., Drawl B., Fox G.R., Gibbons B.J., Trolier-McKinstry S. (2015). Control of Crystallographic Texture and Surface Morphology of Pt/TiO_2_ Templates for Enhanced PZT Thin Film Texture. IEEE Trans. Ultrason. Ferroelectr. Freq. Control..

[15] Borman T., Ko S., Mardilovich P., Trolier-McKinstry S. (2017). Development of Crystallographic Texture in Chemical Solution Deposited Lead Zirconate Titanate Seed Layers. J. Am. Ceram. Soc..

[16] Borman T., Zhu W., Wang K., Ko S., Mardilovich P., Trolier-McKinstry S. (2017). Effect of Lead Content on the Performance of Niobium-doped {100} Textured Lead Zirconate Titanate Films. J. Am. Ceram. Soc..

[17] Dangi A., Cheng C.Y., Agrawal S., Tiwari S., Datta G.R., Benoit R.R., Pratap R., Trolier-McKinstry S., Kothapalli S.R. (2020). A Photoacoustic Imaging Device Using Piezoelectric Micromachined Ultrasound Transducers (PMUTs). IEEE Trans. Ultrason. Ferroelectr. Freq. Control..

[18] Yi G., Wu Z., Sayer M. (1988). Preparation of Pb(Zr, Ti)O_3_ Thin Films By Sol Gel Processing: Electrical, Optical, and Electro-Optic Properties. J. Appl. Phys..

[19] Assink R.A., Schwartz R.W. (1993). Proton and Carbon-13 NMR Investigations of Lead Zirconate Titanate (Pb(Zr, Ti)O_3_) Thin-Film Precursor Solutions. Chem. Mater..

[20] Olding T., Leclerc B., Sayer M. (1999). Processing of Multilayer PZT Coatings for Device Purposes. Integr. Ferroelectr..

[21] Schwartz R.W. (1997). Chemical Solution Deposition of Perovskite Thin Films. Chem. Mater..

[22] Tuttle B.A., Schwartz R.W. (1996). Solution Deposition of Ferroelectric Thin Films. MRS Bull..

[23] Schwartz R., Boyle T., Lockwood S., Sinclair M.B., Dimos D., Buchheit C.D. (1994). Sol-gel Processing of PZT Thin Films: A Review of The State-of-the-Art and Process Optimization Strategies. Integr. Ferroelectr..

[24] Lockwood S.J., Schwartz R.W., Tuttle B.A., Thomas E.V. (1993). Solution Chemistry Optimization of Sol-Gel Processed PZT Thin Films. MRS OPL.

[25] Shepard J.F., Moses P.J., Trolier-McKinstry S. (1998). The Wafer Flexure Technique for the Determination of the Transverse Piezoelectric Coefficient (d_31_) of PZT Thin Films. Sens. Actuators A Phys..

[26] Dangi A., Pratap R. (2017). System Level Modeling and Design Maps of PMUTs With Residual Stresses. Sens. Actuators A Phys..

[27] Jiang X., Lu Y., Tang H., Tsai J., Ng E., Daneman M., Boser B., Horsley D.A. (2017). Monolithic Ultrasound Fingerprint Sensor. Nature.

[28] Balasingam J.A., Swaminathan S., Emadi A. A Low-Frequency Piezoelectric Micromachined Ultrasonic Transducer based on Multi-User MEMS Process with Enhanced Output Pressure. Proceedings of the 2020 IEEE International Ultrasonics Symposium (IUS).

[29] Lysne P.C. (1972). Dielectric Breakdown of Shock-Loaded PZT 65/35. J. Appl. Phys..

[30] Cheng C. (2021). Piezoelectric Micromachined Ultrasound Transducers Using Lead Zirconate Titanate Films. Ph.D. Thesis.

[31] Akkopru-Akgun B., Zhu W., Randall C.A., Lanagan M.T., Trolier-McKinstry S. (2019). Polarity Dependent DC Resistance Degradation and Electrical Breakdown in Nb Doped PZT Films. APL Mat..

[32] Dahl-Hansen R.P., Tyholdt F., Gjessing J., Vogl A., Wittendorp P., Vedum J., Tybell T. (2020). On the effect of Water-Induced Degradation of Thin-Film Piezoelectric Microelectromechanical Systems. J. Microelectromech. Syst..

